# Creating a population-averaged standard brain template for Japanese macaques (*M. fuscata*)

**DOI:** 10.1016/j.neuroimage.2010.05.006

**Published:** 2010-10-01

**Authors:** M.M. Quallo, C.J. Price, K. Ueno, T. Asamizuya, K. Cheng, R.N. Lemon, A. Iriki

**Affiliations:** a Laboratory for Symbolic Cognitive Development, RIKEN Brain Science Institute, Wako, Japan; b Sobell Department, Institute of Neurology, University College London, London, UK; c Wellcome Trust Centre for Neuroimaging, Institute of Neurology, University College London, London, UK; d fMRI Support Unit, RIKEN Brain Science Institute, Wako, Japan; e Laboratory for Cognitive Brain Mapping, RIKEN Brain Science Institute, Wako, Japan

## Abstract

A number of modern digital anatomy techniques, based on structural MR brain images, have recently become applicable to the non-human primate brain. Such voxel-based quantitative techniques require a species-specific standardized brain template. Here we present a brain template for the Japanese macaque (*Macaca fuscata*). The template was designed to be used as a tool for spatially normalising Japanese macaque brains into a standard space. Although this species of macaque monkey is widely used in neuroscience research, including studies of higher cognitive brain functions, no standard MRI template of its brain is presently available. The template presented here is based on T1/T2* weighted, high-resolution 4 T MR images obtained from 16 male adult Japanese macaque monkeys. T1/T2* images were used to correct the signal inequalities resulting from the use of a surface coil. Based on these images, population-averaged probability maps were created for grey matter, white matter and cerebrospinal fluid. The new template presented here should facilitate future brain research using the Japanese macaque monkey. Whole brain templates are available at http://brainatlas.brain.riken.jp/jm/modules/xoonips/listitem.php?index_id=9.

## Introduction

Macaque monkeys represent the best available invasive model for studying the cognitive capacities of the human brain. There is a good correspondence between the brain structures of human and macaque (Orban et al., 2004). Japanese macaques (*Macaca fuscata*) are the commonly used non-human primates for empirical studies of higher cognitive, sensory and motor functions (Isa et al., 2009). A recent literature search reveals that something in the order of 150 studies in the field have been published in the last 10 years. Although the Japanese macaque is valued for its characteristically gentle demeanour and cooperative temperament, which makes it suitable for studies which require extensive training on demanding tasks, a standard MRI template is not currently available. Instead, researchers are obliged to use the template of a related but different species, the Rhesus macaque (*Macaca mulatta*) (McLaren et al., 2009). However, recent advances in precise voxel-by-voxel analysis of structural (McLaren et al., 2010; Quallo et al., 2009), as well as functional brain images (Peeters et al., 2009) require a species-specific template, because subtle differences between the brain structures of different macaque species could be of importance in cognitive studies. Currently there seems to be a preference in neurophysiological studies for males and a review of the use of Japanese macaques from samples of the Kyoto University Primate Research Institute Library Database revealed that 45 males, 20 females and 114 animals of unspecified sex were used in recent physiology and anatomy studies. Therefore, we have established for the first time a population-averaged standardized template of the male Japanese monkey brain.

An important stage in the development of MRI multi-subject analyses was the creation of a human brain template in a standardized coordinate space (Mazziotta et al., 2001; Mazziotta et al., 1995). The recent development of voxel-based digital neuroanatomy, including techniques such as voxel-based morphometry (VBM) and diffusion-tensor imaging (DTI) require such a standardized template in order to transform the raw MRI signal into relative probabilities for different tissue types (i.e. grey matter, white matter and cerebrospinal fluid). These approaches were first established for the human brain (Ashburner and Friston, 2000), and are now becoming available for non-human primate brains for studying fundamental information of higher brain functions (McLaren et al., 2009; Paxinos et al., 2008).

It has been recognized that there are differences between the detailed brain structures of different species of macaque monkeys (Van Der Gucht et al., 2006). These subtle structural differences, such as the shape and depth of sulci, could become crucial in investigations using techniques such as voxel-based-morphometry, and highlight the need for species-specific brain templates. Stereotaxic atlases are available for a number of different macaques, including the Rhesus monkey (*M.* *mulatta*) (Paxinos et al., 2008; Saleem and Logothetis, 2007; Snider and Lee, 1961), pig-tailed monkey (*Macaca nemestrina*) (Cannestra et al., 1997; Winters et al., 1969) and cynomolgus monkey (*Macaca fascicularis*) (Martin and Bowden, 1996). None is currently available for *M. fuscata*. In the case of the Rhesus monkey (*M.* *mulatta*), modern atlases combine information from histological cytoarchitectonic sections from a single subject, combined with structural MRI data from the same animal (Paxinos et al., 2008; Saleem and Logothetis, 2007). In addition, a population-averaged standard template that is aligned to the stereotaxic atlas has recently been created for the Rhesus monkey (McLaren et al., 2009). Population averaged MRI templates are also available for the pig-tailed macaque (Black et al., 2001a) and the baboon black (Black et al., 2001b). However, despite the availability of sample MRI images of the Japanese macaque (AIST-RIO-DB, 2006), a complete brain template for this species is not yet available, even though it is the subject of many studies of higher cognitive functions. The publication of a template for the Japanese macaque brain is clearly long overdue, since pioneering MR studies using this species (Hayashi et al., 1999; Nakahara et al., 2002). Here we present a template for the Japanese macaque and in doing so we illustrate the procedures necessary to create a template for other species, as described (Ashburner, 2007). The template described here is for the registration of MRI images of *M. fuscata* to a common space to enable the analysis of grouped imaging data. Although we do provide images aligned to standard stereotaxic coordinates, we are not attempting to create a stereotaxic or histological atlas for this species.

## Methods

### Monkeys

16 adult *M. fuscata* monkeys were scanned. These included 16 males with weights ranging from 4.1 to 10.2 kg (mean 7.1 kg; median 8.1 kg). This study was approved by the local Animal Experiment Committee and was conducted in accordance with the Guidelines for Conducting Animal Experiments of the RIKEN Brain Science Institute.

### Structural MRI

**Fig. S1:**
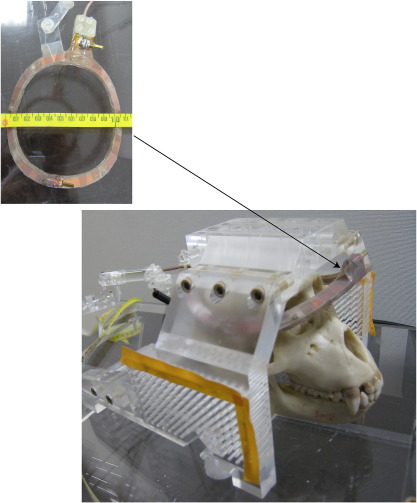
Photograph showing the coil (above) and the coil within the headholder (below). The head is secured within the headholder with ear bars in the external auditory meatus and eye bars on the infraorbital margin. Vitamin E-filled glass capillary tubes can be mounted in the array of calibrated holes in the top and side to be used as stereotaxic markers. The coil (105 mm in diameter) is attached to the headholder through drilled holes at the top the headholder.

**Fig. S2:**
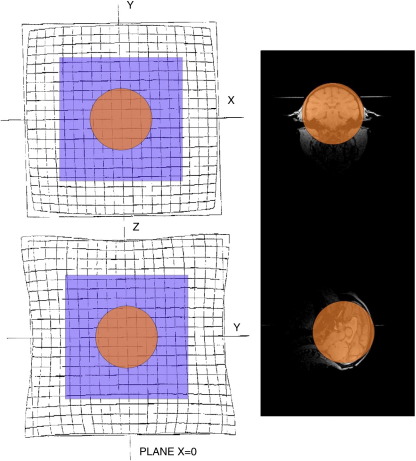

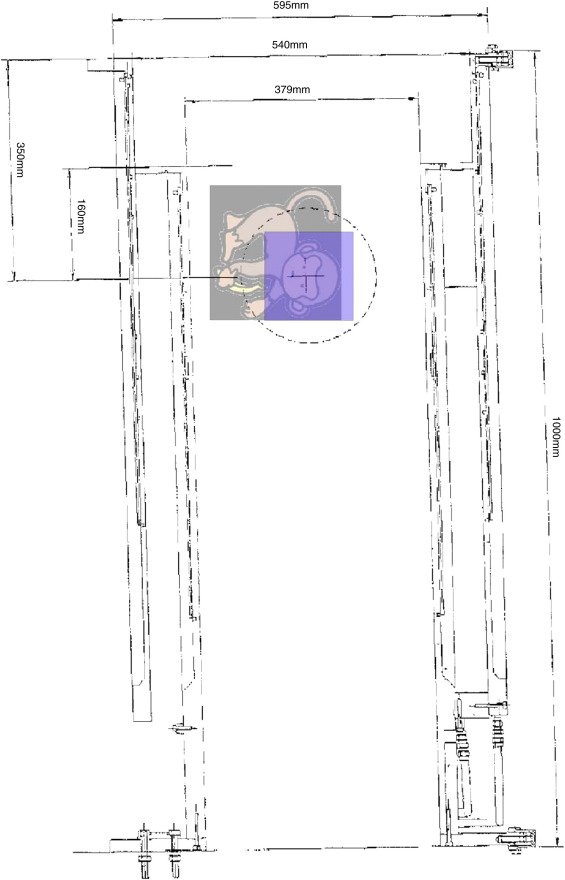
A distortion map showing the possible areas of distortion. The monkey's head was positioned centrally in the region of minimal distortion.

Prior to MRI scanning, monkeys were deeply anesthetized with ketamine/Domitor. The intramuscular injection was a mixture made up of 1.1 ml ketamine (50 mg ml^−^^1^ ketamine hydrochloride; Daiichi Sankyo Co., Ltd; Tokyo, Japan) and 0.8 ml Domitor (1 mg ml^−^^1^ medetomidine hydrochloride; Nippon Zenyaku Kogyo Co., Ltd; Fukushima, Japan). The combined dose given was ketamine 0.08 mg kg^−^^1^ i.m. and 0.11 Domitor mg kg^−^^1^ i.m. Once anesthetized, monkeys were transferred to a plastic stereotaxic headholder (Shimaere, Ltd. Tokyo, Japan) and the head secured with ear bars in the external auditory meatus and eye bars on the infraorbital margin. The headholder carried an array of calibrated holes drilled in its top and side. Vitamin E-filled glass capillary tubes (outside diameter 2 mm) were mounted in these holes and used as stereotaxic markers. Structural MRI scans were obtained using a 4 T Varian Unity Inova MR scanner (Varian NMR Instruments, Palo Alto, CA) with a 3 in single loop surface coil (Takashima Seisakusho Ltd.; Tokyo, Japan). Photographs of the coil and headholder are shown in Supplementary Fig. 1. The coil was large and was positioned close to monkey's head, which it encompassed; as a result any distortion of the images should have been minimal (see distortion map in Supplementary materials, Fig S2). For each scan, both a T1- and a T2*-weighted images were obtained, and the images were combined to provide a T1/T2* image. T1-weighted images were acquired with a magnetization-prepared 3D FLASH sequence (time of repetition 13 ms, echo time 3.8 ms, time to inversion 0.5 s, flip angle 11°, matrix size 256 × 256 × 256, and field of view 12.8 × 12.8 × 12.8 cm) and had a voxel size of 0.5 × 0.5 × 0.5 mm. T2*-weighted images were acquired with the same parameters, but without inversion recovery.

**Fig. 1:**
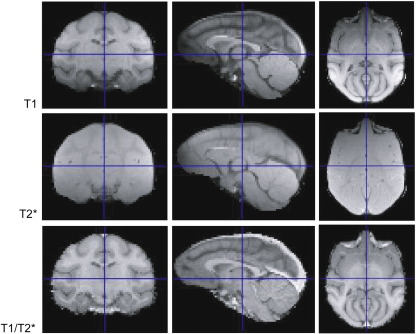
T1, T2* and T1/T2* images from a single monkey are shown. The first row illustrates coronal; sagittal and horizontal sections of the T1 images. T2* and T1/T2* images are shown on the second and third rows, respectively.

Due to the use of a high-magnetic field (4 T) scanner to obtain high spatial resolution (voxel size 0.5 × 0.5 × 0.5 mm) images, we used a surface emission coil rather than a volume (‘bird cage’) coil. This procedure obliged us to sacrifice equal spatial intensity distribution, depending on the placements of the coil for both T1 and T2* weighted images as depicted in the top, and middle rows of Fig. 1, respectively. To compensate for this spatial bias, voxel-by-voxel calculation of the ratio (T1/T2*) of both images were calculated to equalize signal density across a large field (Mugler and Brookeman, 1990). This procedure not only equalized the signal strength but also produced images with high contrast between the gray matter, white matter and cerebrospinal fluid (CSF) as depicted in the lower row of Fig. 1.

## Template creation

### Skull-stripping and realigning

Images were skull-stripped using BET [Brain Extraction Tool] (Smith, 2002) in FSL [Functional MRI of the brain Software Library] (Analysis Group, FMRIB, Oxford, UK). The remaining steps were carried out in SPM5 (Wellcome Trust Centre for Neuroimaging, UCL Institute of Neurology, London, UK) running under Matlab (MathWorks, Natick, MA). The skull-stripped images were then realigned to one of the 16 images using the automated co-register step in SPM5.

### Segmentation of images and creation of a standard template

**Fig. 2:**
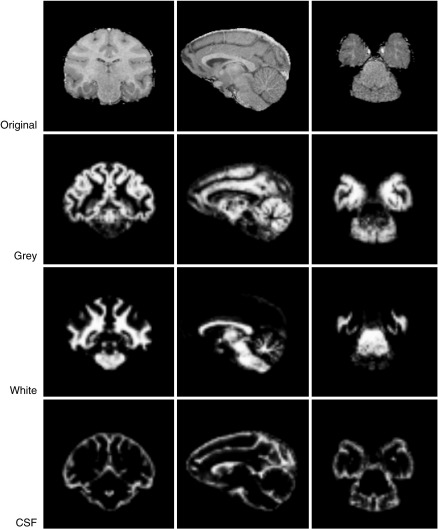
The original images followed by the grey, white and CSF segmented images (coronal; sagittal and horizontal sections) from a single monkey are shown on the first, second, third and fourth rows respectively.

**Fig. 3:**
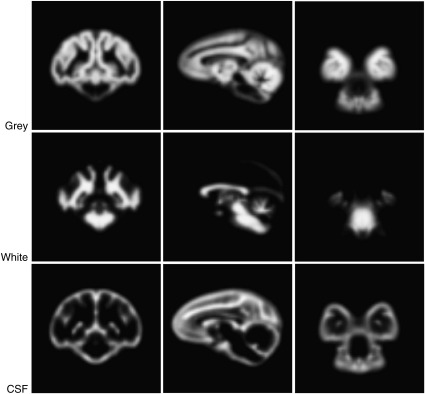
Coronal, sagittal and horizontal sections of the T1/T2* grey, white and CSF template are shown. Grey, white and CSF are on the first, second and third rows, respectively.

A two stage process was used to segment the realigned images into grey matter, white matter and CSF. First, we created tissue probability maps for the Japanese macaque using the standard procedures in SPM5 with the 112RM-SL (McLaren et al., 2009) probability maps for the Rhesus macaque. In an earlier version of the template, we used human tissue probability maps resized to 75% of their original size, and the resulting template showed only minor differences from that derived from the monkey probability map. We opted for no affine regularisation and a sampling distance set to 1 mm. Examples of the results from an individual monkey are illustrated in Fig. 2. In the second step, we used the DARTEL [Diffeomorphic Anatomical Registration Through Exponentiated Lie algebra] tool box in SPM5 (Ashburner, 2007). The first step was an initial import of the segmented data which produced rigidly aligned tissue class images for each animal. These rigidly aligned images were then used to create a template; a Gaussian smoothing kernel of 3 mm was applied to the template. To suppress any bias towards the Rhesus macaque template we iteratively (i) generated a template image from the segmented images of all monkeys and (ii) registered each image to this template so that an updated template could be generated. This process continued iteratively, with each stage beginning with the previously created template, resulting in a series of templates, the last of which was an average of all the DARTEL registered data. Fig. 3 shows each of the templates for grey matter, white matter and CSF. Whole brain templates are available at http://brainatlas.brain.riken.jp/jm/modules/xoonips/listitem.php?index_id=9.

### Application of tissue priors to a T1w image

**Fig. 4:**
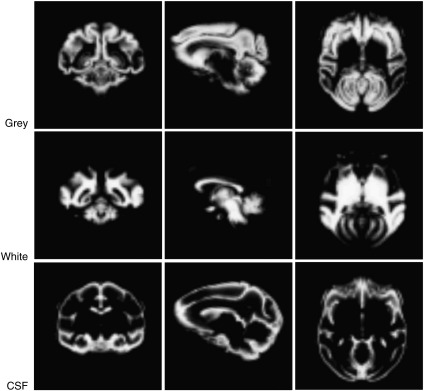
Coronal, sagittal and horizontal sections of the T1 grey, white and CSF segmentations are shown. Grey, white and CSF are on the first, second and third rows, respectively.

Since most imaging studies use T1 images, a template for T1 images was required. In order to produce this, we segmented and normalised a T1 scan from one monkey to the template using our new templates as tissue probability maps. The chosen image was from a monkey whose T1/T2* scans had already been included in the template. The T1 image is, however, different, and has more susceptibility artefacts due to the unequal spatial intensity distribution caused by the surface coil. This image was successfully segmented and normalised, demonstrating that the T1/T2* template can be used to normalise and segment images susceptible to artefacts. Fig. 4 shows the resulting segmented images from a single animal.

### Brain images registered to stereotaxic coordinate system: comparison with Rhesus macaque

**Fig. S3:**
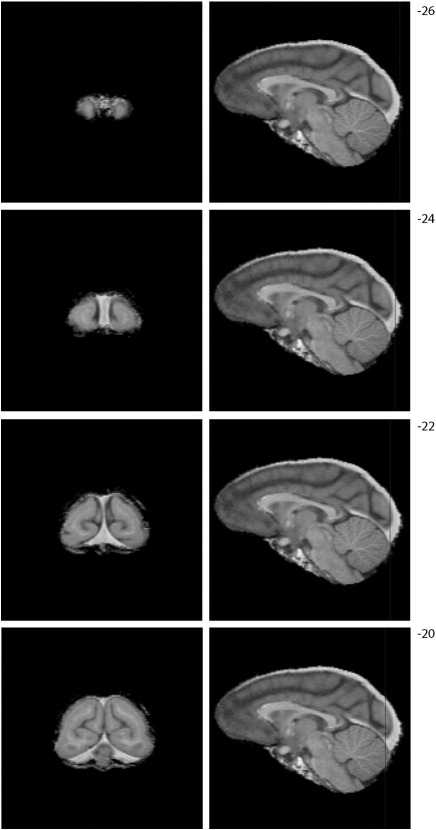

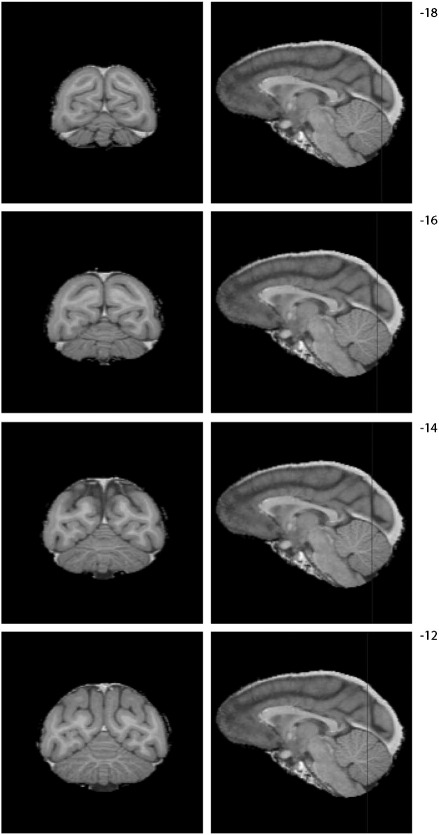

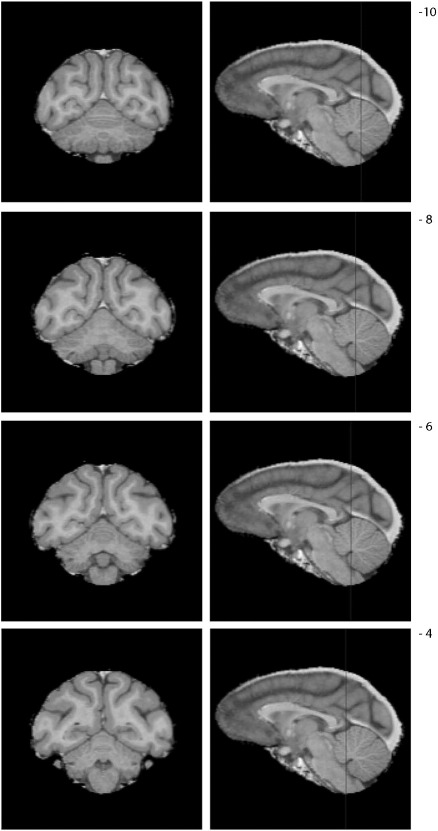

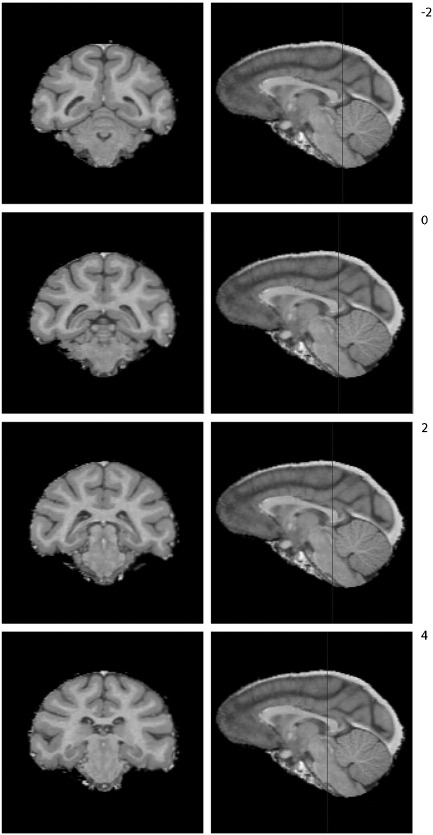

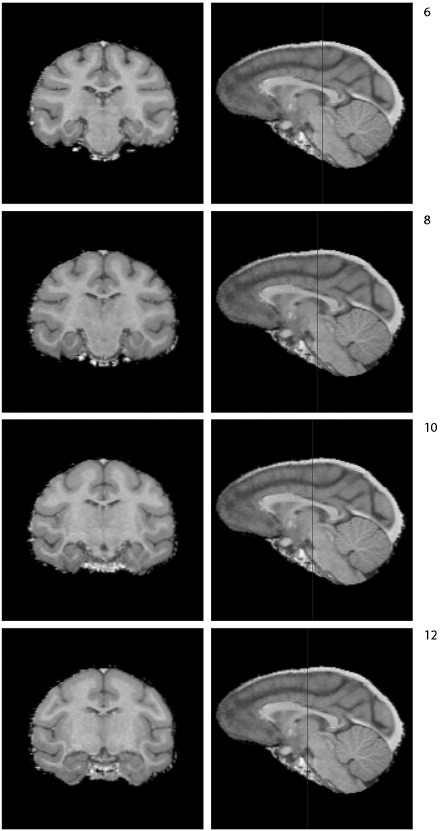

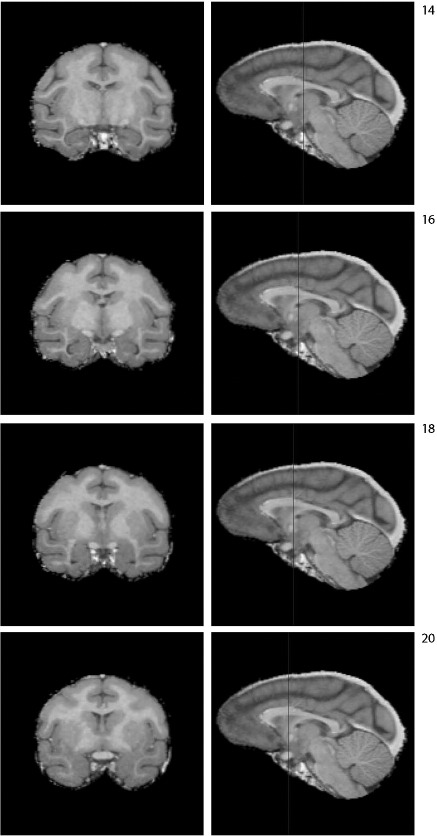

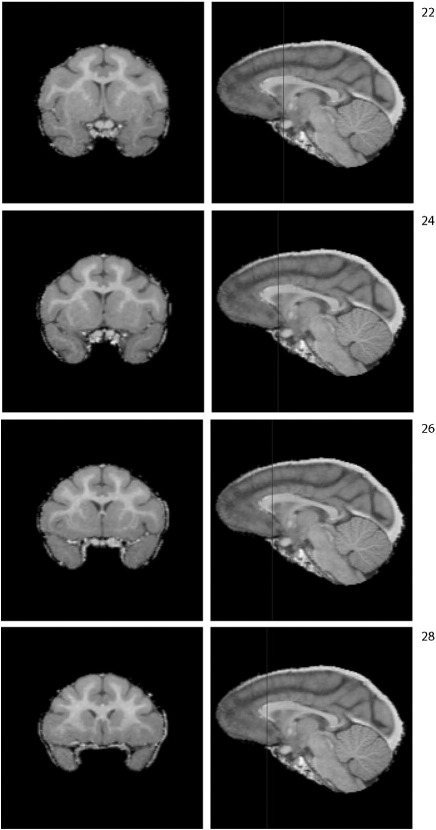

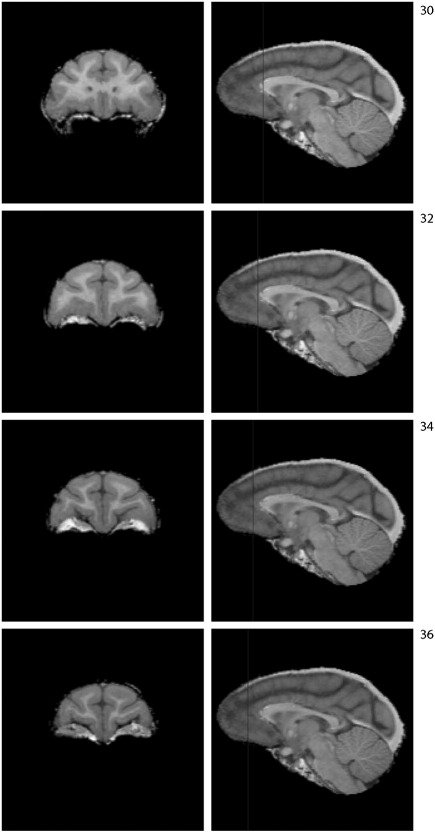

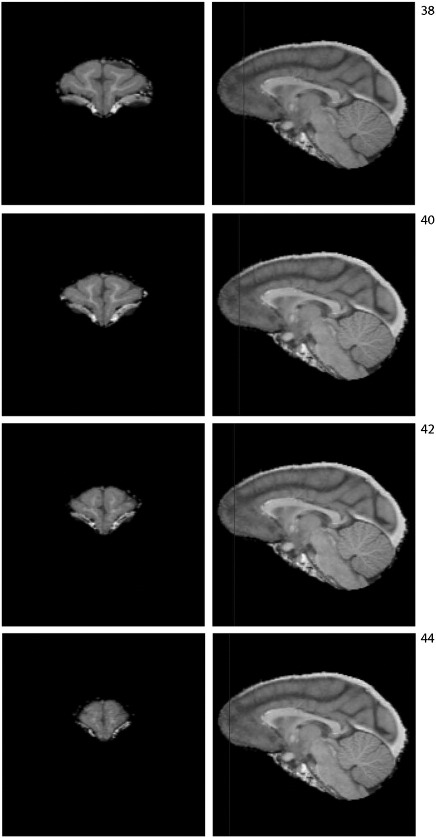

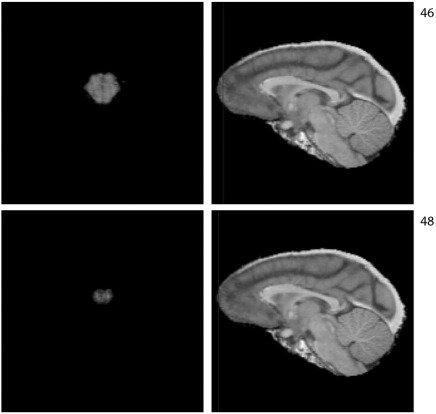
A series of brain images registered to stereotaxic coordinate system in the coronal plane. Images are provided from AP0 to A+ 48 and to P− 26, in 2 mm steps. Coronal slices are shown on the left. The black line in the sagittal section (on the right) indicates the location of the section.

Since there is currently no stereotaxic atlas of the *M. fuscata* brain, it was not possible to relate the brain template to stereotaxic measures. In order to show representative sections in standard stereotaxic space, the stereotaxic coordinates were determined for three brain landmarks in each of the 16 monkeys. These landmarks were the anterior and posterior commissures, both measured at the midline, and the rostral pole of the right caudate nucleus. All measurements were made with reference to the stereotaxic markers visible in the unstripped T1/T2* images from each of the 16 monkeys (see Methods). This table shows substantial variation across the sample, with a range of up to 12 mm for some measurements. The mean (± SD) *x*, *y* and *z* coordinate values are shown in Table 1. The mean values were then used to calculate the stereotaxic zero for the brain template. Sample coronal and saggital sections, based on the template, are presented in Supplementary materials (Fig. S3). These are based upon T1/T2* images from the brain of one monkey that had been normalised to fit the template. Images are provided from AP0 to A+ 48 and to P− 24, in 2 mm steps. All images are registered to AP0.

Table 1 also includes the coordinates for the same three landmarks derived from the 112RM-SL template (McLaren et al., 2009). There is overall a good correspondence between the mean values of the three core brain structures from our Japanese macaque template and the published data for the same structures in the Rhesus macaque, although some values were as much as 3 mm different. Since stereotaxic coordinates are based on bony landmarks, this may reflect differences in skull shape. Inspection of the coordinates of a number of sulcal landmarks (including a principal, central, intraparietal and calcarine sulci), again suggested variations of 3 to 4 mm.

### Registration of key brain landmarks within the template: comparison with Rhesus macaque

A more direct approach, which excludes differences in stereotaxic coordinates due to differences in skull shape, is to compare the relative distance between brain landmarks. We measured the positions of a number of different landmarks, including cortical sulci, relative to the coordinates of the anterior commissure (AC; cf McLaren et al., 2009); these relative brain measurements are shown in Table 2. We compared these values with those of the same landmarks in the Rhesus macaque (112RM-SL template). Again, in general, the measurements relative to AC were in good agreement with those from the Rhesus macaque, although once again there were differences of up to 2–3 mm.

## Discussion

We have created a standardized brain template for the Japanese macaque, which is a common model used for studies of higher cognitive, sensory and motor functions. The template will be of particular use in the registration of MR images using non-invasive brain imaging. The procedures described here could also be applied to produce specific templates for other experimental animals.

The template presented here was created using DARTEL. An alternative method of template creation involves registering all the images to a common reference subject and generating an average of these registered images. This results in a template that is biased towards to the original reference image. Using DARTEL removes this bias by iteratively registering each images to an average of the rigidly aligned images (Ashburner and Friston, 2009).

The 112RMSL Rhesus macaque template (McLaren et al., 2009) used 112 monkeys to ensure they captured the extent of variability in brain structure within the species. The number of animals in our template (16) is less than in the 112RM-SL Rhesus macaque template but similar to other species-specific non-human primate templates: 9 monkeys were used to create the baboon template (Black et al., 2001b) and the pig-tailed macaque template is based on 12 monkeys (Black et al., 2001a). To maximise the generalizability of our template, we included monkeys with a wide range of body weights (4.1–10.2 kg). In addition, all 16 monkeys were scanned under identical conditions in the same MR scanner.

Since we used only male monkeys, the data are biased towards the males, however as mentioned above, males are more commonly used in non-human primate research than females. Other non-human primate templates also show a male bias; in the McLaren et al. (2009) study 82 male Rhesus macaques are used compared to 32 females; the baboon template is based on 9 males and 3 males (Black et al., 2001b) and the pig-tailed macaque is based on 12 males only (Black et al., 2001a). Additionally, this method could be used to create a female template and both templates could be used to compare male/female characteristics.

To be able to create a reliable template based on the structure of a number of different monkeys, we had to be able to segment images of high resolution from each monkey. For this we employed at high field (4 T) scanner and a large, close-fitting surface emitting coil (Fig. S1), which produced excellent images, with minimal distortion (Fig. S2).

Due to the use of a surface coil, these images were rather non-uniform across the whole image field, and this could have compromised the process of segmenting the images, as this required a high level of contrast between grey and white matter and CSF. By using the T1/T2* images the non-uniformity of MRI signal strength across the whole image field, due to uneven magnetic field was very effectively compensated (Van de Moortele et al., 2009). We were able to obtain excellent quality images of high spatial resolution and with sharp contrast between grey and white matter. Based on these images, we were able to produce tissue probability maps for grey matter, white matter and CSF from the T1/T2* images, and could also use these to create a template and probability maps for the T1 images.

Our investigation suggests that there are only minor differences between Rhesus and Japanese macaques in the both stereotaxic and relative measures of selected brain landmarks including the anterior and posterior commissures; caudate nucleus and central sulcus (Tables 1 and 2). Therefore, although differences between various macaque species are probably subtle, differences may still exist between species in detailed macroscopic structures, such as the shape and depth of cortical sulci and gyri (Van Der Gucht et al., 2006). This new brain template will contribute to the quantitative description of species differences in future investigations.

Although in the final version of this template, we used the Rhesus macaque template (McLaren et al., 2009) as the starting point for the segmentation, we also generated a template using priors from the human tissue probability maps after resizing these maps to 75% of their original size. The resulting template showed only minor differences from that derived from the monkey probability map. This suggests that, if a template is not already available for the same species, it is possible to use tissue probability maps that are available from the most similar animal. An alternative approach would be to use FAST (FMRIB's Automated Segmentation Tool) in FSL.

The present database would also be useful for other possible applications in which accurate registration of the individual data to a species-specific template is required, such as for voxel-based morphometry (VBM) analyses (McLaren et al., 2010; Quallo et al., 2009), for accurate electrode tip placements both for recording and stimulation of the brain, and for three dimensional registration of fMRI data using surface brain maps (Van Der Gucht et al., 2006; Van Essen and Dierker, 2007).

## Figures and Tables

**Table 1:** Stereotaxic coordinates of brain landmarks in *M. fuscata*. Mean, minimum, maximum and SD of the coordinates of brain landmarks derived from 16 individual *M. fuscata* monkeys. The landmarks were the anterior and posterior commissures, both measured at the midline, and the rostral pole of the right caudate nucleus. All measurements are in mm and are referenced to ear bar zero, and were made with reference to the stereotaxic markers visible in the unstripped T1/T2* images. Measurements derived from *M. mulatta* described in the 112RM-SL template (McLaren et al., 2009) are included in the table. Differences between the mean *M. fuscata* and *M. mulatta* values are highlighted by asterisks (* > 1 mm; ** > 2 mm; *** > 3 mm).

	Anterior commissure			Posterior commissure			Rostral pole, caudate n.		
*M. fuscata*	*X*	*y*	*z*	*x*	*y*	*z*	*x*	*Y*	*z*
Mean	0.0	19.9	15.1	0.0	6.1	14.8	5.8	29.5	22.3
Min	0.0	13.3	13.3	0.0	0.0	12.6	4.6	22.9	20.2
Max	0.0	24.7	17.7	0.0	9.6	17.9	7.6	34.5	26.2
± SD	0.0	3.0	1.6	0.0	2.4	1.5	0.9	3.0	1.6
*M. mulatta*	0.0	21.0*	12.0***	0.0	7.5*	14.0	6.5	33.0***	17.5***

**Table 2:** Relative locations of brain landmarks in the *M. fuscata* brain: comparison with *M. mulatta*. Coordinates of brain landmarks in Japanese macaque in comparison with 112RM-SL Rhesus monkey template (McLaren et al., 2009). All measurements are in mm and are referenced to the anterior commissure (AC). The asterisk highlights any differences between the species * > 1 mm; ** > 2 mm and *** > 3 mm.

Landmark	Rhesus macaque template			Japanese macaque template		
*x*	*y*	*Z*	*x*	*Y*	*z*
AC	0	0	0	0	0	0
PC	0	−13.5	2	−0.2	−14.2	0.4*
L ant. caudate	−6.5	12	5.5	−6.8	9.3**	9.1***
R ant. caudate	6	12	5.5	6.4	9.4**	9.1***
L lat. central S	−24.5	−4.5	8.5	−22.3**	−4.9	7.1*
R lat. central S	23.5	−4	8.5	23.7	−5.5*	6.2**
L med. central S	−18	−6	13.5	−15.5**	−7.1*	12.3*
R med. central S	18	−4.5	13	16.3*	−7.1**	12.1
